# Probing Side-Chain Engineering for Modulating Exciton Dynamics in Non‑fullerene Acceptors

**DOI:** 10.1021/acsomega.5c08337

**Published:** 2025-11-18

**Authors:** Sanyam Jain, M Sridevi, Tanushree Majhi, Narendra Pratap Tripathi, Saurabh Kumar Saini, Anita Kumari, Sanchita Sengupta, Rajiv K. Singh

**Affiliations:** † Photovoltaic Metrology Section, Advanced Materials & Device Metrology Division, 548682 CSIR-National Physical Laboratory, Dr K. S. Krishnan Marg, New Delhi 110012, India; ‡ Academy of Scientific and Innovative Research (AcSIR), Ghaziabad 201002, India; § Department of Chemical Sciences, 124268 Indian Institute of Science Education and Research (IISER), Mohali, Knowledge City, Sector 81, Punjab 140306, India; ∥ Department of Physics, Indian Institute of Technology Roorkee, Roorkee, Uttarakhand 247667, India

## Abstract

The last 2 years have witnessed rapid progress in organic solar cells (OSCs), and precise modulation of frontier orbital energies without compromising light absorption is crucial for optimizing organic semiconductors. This study presents a predictive side-chain engineering approach for non-fullerene acceptors (NFAs) using quantum-chemical calculations to identify substituents that selectively shift the HOMO and LUMO levels while preserving the optical bandgap. Guided by structural and excited-state simulations, two non-fullerene acceptors, PDIEH and PDIIN, were designed, incorporating flexible 2-ethylhexyl and rigid indanyl groups, respectively. Electronic structure modeling revealed that flexible alkyl groups stabilize the electronic structure via inductive effects, whereas rigid aromatic groups introduce partial conjugative perturbations, resulting in distinct energy level shifts. Despite similar optical gaps (∼2.27 eV), these electronic modulations critically impact exciton dynamics as revealed by ultrafast transient absorption spectroscopy (UTAS). PDIEH exhibits deeper LUMO levels and prolonged charge-separated lifetimes, while PDIIN shows faster recombination. This study establishes a chemically intuitive framework where side-chain engineering enables predictive control of electronic properties and charge behavior in organic photovoltaics.

## Graphical abstract

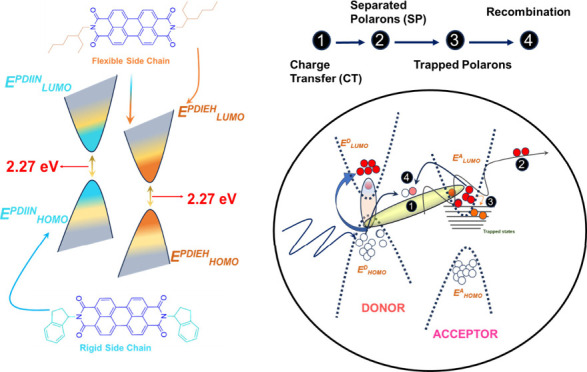

## 1 Introduction

Organic solar cells (OSCs) have garnered immense interest in next-generation photovoltaics due to their potential for lightweight,^1,2^ flexible, and semitransparent energy-harvesting applications.^3,4^ Their compatibility with low-temperature, roll-to-roll fabrication techniques renders them ideal for scalable and cost-effective production.^5,6^ Recent advancements in material design have propelled OSC power conversion efficiencies (PCEs) beyond 19%, primarily driven by innovations in donor–acceptor molecular systems.^7–10^ OSCs demand precise donor and acceptor molecular properties tuning to achieve optimal energy level alignments, facilitating efficient exciton dissociation and charge extraction. While extensive molecular engineering of donor–acceptor systems has improved power conversion efficiencies, a systematic methodology that directly links molecular structure to photophysical behavior remains underdeveloped.^11–13^ Conventional approaches modifying the conjugated backbone often disturb absorption profiles. Side-chain engineering offers a subtler strategy; wherein electronic effects such as inductive stabilization or conjugative interaction can fine-tune frontier orbitals without altering the π-conjugated system responsible for light absorption.^14,15^ Rational side-chain modification strategies for non-fullerene acceptors (NFAs) that control charge dynamics at ultrafast time scales are unexplored. Among NFAs, small-molecule perylene diimide derivatives (PDI) stand out for their strong visible-light absorption, high electron affinity, and robust electron transport properties.^16,17^ However, their practical application is often hindered by uncontrolled π–π stacking and excessive aggregation, resulting in suboptimal film morphology and inefficient charge separation.^18^

In our study, Quantum-chemical simulations are employed as a predictive tool to preselect side-chain substituents that modulate absolute energy levels while preserving optical bandgaps.^19,20^ However, the calculated frontier molecular orbitals (FMOs), energy levels, electron density distributions, and singlet–triplet transitions provided critical insights into the photophysical behavior and charge transport potential of both PDI derivatives.^21,22^ Computational results have been obtained that indicate deeper FMOs such as HOMO (−5.98 eV) and LUMO (−3.45 eV) levels for PDIs derivatives, which guided the synthesis by identifying promising electronic characteristics prior to material preparation. The synthesis of the two PDI-based NFAs, designated as PDIIN (with indanyl substituents) and PDIEH (with 2-ethylhexyl substituents), was carried out using a straightforward imidization reaction starting from perylene-3,4,9,10-tetracarboxylic dianhydride (PTCDA).^23^ In PDIEH, flexible 2-ethylhexyl groups were incorporated with minimal electronic interaction, while PDIIN featured rigid indanyl groups capable of weak π-conjugative interaction. Theoretical modeling and experimental analyses confirmed that side-chain-induced effects precisely shifted the HOMO and LUMO levels without altering the optical transition energies. The materials were blended with P3HT (P3HT:PDIEH and P3HT:PDIIN) and PTB7 (PTB7:PDIEH and PTB7:PDIIN) in an equal ratio. These mixtures were then subjected to a comprehensive suite of characterization techniques to evaluate their structural integrity, optical properties, electrochemical behavior, and excited-state charge dynamics. It was revealed that PDIEH exhibits higher charge stability (∼9.84 ns), while faster charge transfer (∼0.41 ps) and slower recombination (∼6.59 ns) were observed in blends of PDI, with PTB7. Although both materials have experimental optical gaps close to 2.27 eV, the electronic characteristics were shown to play a crucial role in exciton dynamics,^24,25^ as demonstrated by ultrafast transient absorption spectroscopy (UTAS). Deeper LUMO levels and longer lifetimes for charge separation were found in PDIEH, whereas more rapid recombination was demonstrated by PDIIN. This study aims to explore PDI-based NFAs through quantum-chemical simulations, which have successfully demonstrated the impact of side-chain composition on electronic properties and exciton dynamics for advanced optoelectronics applications and photovoltaic generation.

## 2 Materials and Characterization Techniques

The precursors, perylenetetracarboxylic dianhydride (PTCDA) and amine, i.e., 1-aminoindan and 2-ethylhexyl, were acquired from Sigma-Aldrich (Merck) and utilized without further purification. All reactions were executed in anhydrous solvents using oven-dried glassware under a nitrogen atmosphere. Thin-layer chromatography was employed for real-time monitoring of reactions, and product purification was achieved through column chromatography.

At room temperature, a Bruker Biospin Avance III FT-NMR 400 MHz spectrometer was used to record ^1^H NMR spectra in deuterated chloroform (CDCl_3_) using trimethyl silane (TMS) as the primary reference. This provided detailed insights into the molecular structure. Structural confirmation was conducted via Fourier transform infrared spectroscopy (FT-IR) using a PerkinElmer FT-IR Spectrum 2. The samples, crushed with anhydrous KBr, underwent scanning from 4000 cm^–1^ to 400 cm^–1^, with a background spectrum in air collected before sample scanning.

Cyclic voltammetry measurements were carried out in a dichloromethane solution of PDIIN and PDIEH employing a three-electrode system. The experimental setup consisted of a platinum disc working electrode, a platinum wire counter electrode, and an Ag/AgCl reference electrode. The supporting electrolyte used was 0.1 M TBAPF_6_ (tetra-*n*-butylammonium hexafluorophosphate). The potential was calibrated using ferrocene as an internal standard. All measurements were conducted under a nitrogen atmosphere, with the solution degassed for 15 min before each run. Current (*I*) vs voltage (*V*) curves were recorded using an Autolab electrochemical workstation at a scan rate of 100 mV^–1^ s^–1^. The quantum-chemical simulations (DFT) were executed via the Gaussian 16 package at the B3LYP/6-31G­(d,p) level of theory to find out the frontier molecular orbital energy levels (HOMO–1, HOMO, LUMO, and LUMO+1) for PDIEH and PDIIN.

Photoluminance (PL) was recorded using an FLS1000 scientific spectrophotometer. UTAS experiments were conducted on the thin films deposited onto a quartz substrate, utilizing optical pulses generated by a Ti:sapphire laser amplifier (Micra, Coherent) with parameters of 35 fs duration and 4 mJ/pulse energy operating at a frequency of 1 kHz and emitting light at a wavelength of 800 nm. The laser beam was divided into two components using a 70:30 beam splitter. An optical parametric amplifier (TOPAS-C, Light Conversion) was utilized to adjust the wavelength of the high-intensity beam (pump) across the range of 190 to 2600 nm. A sapphire crystal transformed the low-intensity portion (probe) into a white light continuum. Both the pump and probe beams were carefully aligned to overlap spatially on the sample. The probe beam was temporally delayed for transient measurements relative to the pump beam using a 6 ns delay stage.^26,27^

## 3 Results and Discussion

### 3.1 Theoretical Electronic Structures, Molecular Design, and Synthesis of PDIEH and PDIIN

PDIEH and PDIIN, synthesized using a cost-effective method from a PDI core through imidization reactions with 2-ethylhexan-1-amine and 1-aminoindane, were blended with P3HT (P3HT:PDIEH and P3HT:PDIIN) and PTB7 (PTB7:PDIEH and PTB7:PDIIN) in a 1:1 mixture. Electronic structure calculations and excited state simulations were employed to model the PDI derivatives’ ground-state (S_0_) and excited-state (S_1_, S_2_, T_1_, T_2_, T_3_) electronic structures, depicted in Figure 1a,b. Using the B3LYP/6-31G­(d,p) level of theory in the Gaussian16 suite, the frontier orbital energies (HOMO, LUMO) and electron density distributions that influence charge transport and photophysical behavior were determined. Both PDIEH and PDIIN preserve the characteristic π–π* conjugated framework of the PDI core, as reflected by the spatial localization of the HOMO and LUMO primarily on the central perylene unit. However, the side chains—flexible alkyl groups in PDIEH and rigid aromatic indanyl groups in PDIIN—modulate the PDI scaffold’s electronic environment differently. The flexible 2-ethylhexyl side chains are electronically inert, exhibiting neither conjugative nor hyperconjugative interactions with the core. The inductive (+I) effect in PDIEH slightly stabilizes the electronic system, leading to deeper HOMO (−5.98 eV) and LUMO (−3.45 eV) levels. In contrast, the rigid indanyl substituents engage in partial conjugation through the imide nitrogen linkage, introducing weak electronic communication with the PDI π system. This leads to a minor HOMO destabilization (−5.97 eV) compared to PDIEH, while the LUMO remains unperturbed mainly. Interestingly, despite the shift in absolute energy levels, the optical bandgap (*E*_g_) remains virtually unchanged (∼2.53 eV for both molecules), indicating that the overall π–π* transition energy is preserved. The side-chain substituents influence the relative positioning of the FMOs without significantly altering the intrinsic electronic delocalization across the PDI framework. The electronic structure analysis further reveals the electron density distribution across molecular orbitals. The LUMO and LUMO+1 orbitals are primarily localized on the perylene core, confirming its role as the electron-accepting unit. In contrast, the HOMO and HOMO–1 orbitals show a degree of delocalization influenced by the alkyl and indane groups. Notably, the indane groups in PDIIN contribute slightly to the HOMO, explaining its slightly higher energy compared to PDIEH.

**Figure 1:**
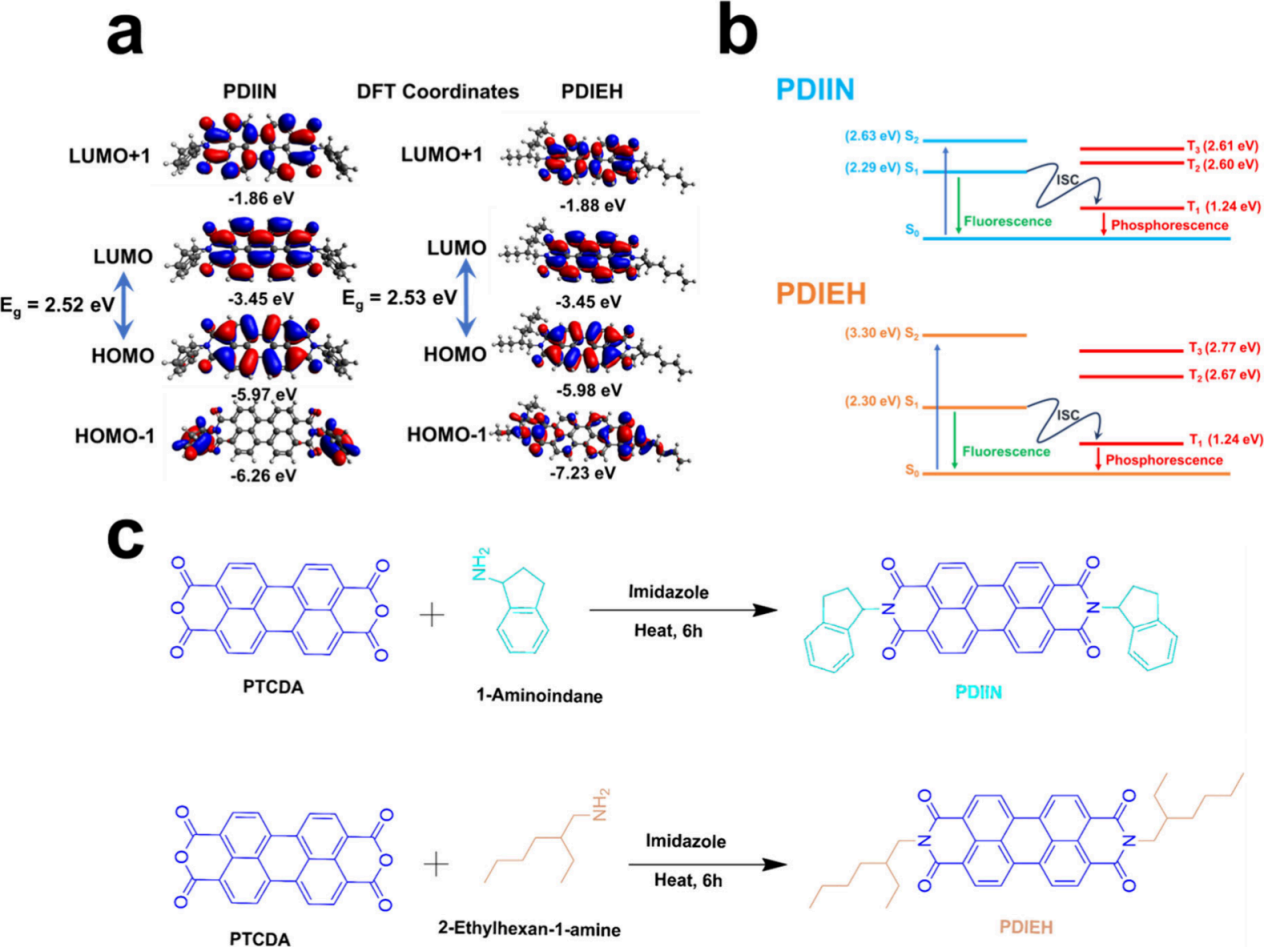
(a) DFT-calculated frontier molecular orbitals (HOMO/LUMO) of PDIEH and PDIIN. (b) Jablonski diagrams showing singlet/triplet transitions and ISC pathways for PDIEH and PDIIN. (c) Schematic representation of the synthesis of PDIEH and PDIIN via imidization of perylene-3,4,9,10-tetracarboxylic dianhydride (PTCDA) with flexible 2-ethylhexyl and rigid indanyl amines, respectively. Side-chain engineering enables systematic modulation of solubility and optoelectronic properties in non-fullerene acceptors.

To estimate the excited-state properties, such as singlet (S_1_, S_2_) and triplet (T_1_) energy levels, excited-state simulations were performed. These parameters were used to construct Jablonski diagrams and interpret radiative (fluorescence, phosphorescence) and nonradiative (internal conversion, intersystem crossing (ISC)) relaxation processes. The S_1_ energies of PDIEH and PDIIN were ∼2.30 eV and ∼2.29 eV, respectively, confirming their capability for visible-light absorption. Both compounds shared similar triplet energies (∼1.24 eV), essential for potential intersystem crossing pathways that influence exciton dynamics and charge carrier lifetimes. The presence of such low-energy triplet states can facilitate processes such as intersystem crossing (ISC) and triplet sensitization, potentially enhancing charge carrier dynamics. The narrow gap between singlet and triplet states indicates efficient singlet exciton dissociation into free charges or triplet excitons, reducing recombination losses. Moreover, PDIEH exhibits a higher-energy S_2_ state at 3.30 eV, enabling it to absorb in the ultraviolet range, thus broadening its spectral response. From the perspective of molecular orbital theory, the deeper energy levels observed in PDIEH are rationalized by a reduction in electronic perturbation, allowing for a more “isolated” core electronic structure to be maintained. In contrast, a slight conjugative interaction in PDIIN results in the HOMO electron density being broadened marginally into the side groups, which raises its energy. This subtle electronic modulation is critically affecting exciton behavior. Deeper LUMO levels in PDIEH create stronger driving forces for electron transfer from donor polymers, while the elevated energy levels of PDIIN may lead to a reduction in electron extraction efficiency.

Guided by electronic structure calculations, side chains were strategically selected to influence orbital energies while maintaining the intrinsic optical gap. The PDI core was functionalized via imidization^28^ reactions with 2-ethylhexan-1-amine and 1-aminoindan, yielding PDIEH and PDIIN, respectively, as depicted in Figure 1c. Including bulky, rigid indanyl side chains in PDIIN was intended to reduce uncontrolled aggregation while preserving conjugation and π–π interactions, crucial for charge mobility.^29,30^ Conversely, the branched 2-ethylhexyl chains in PDIEH were introduced to enhance solubility and film formation, albeit at the cost of reduced molecular planarity and π–π stacking.^31,32^ This modular synthetic approach directly assessed how side-chain structure modulates photophysical and morphological characteristics. The design rationale, supported by DFT/TD-DFT predictions, establishes a robust framework for structure–property relationship studies in NFA materials, setting the stage for subsequent characterization of their optical, electrochemical, and photovoltaic performance. While this enhances miscibility with donor polymers and enables high-quality film formation, the trade-off is a reduction in π–π stacking interactions. This side-chain engineering approach is well-established for tuning NFA aggregation and electronic interaction with donor polymers.^33,34^

A suite of characterization techniques, including NMR (see Figure S1) and FT-IR spectroscopy, was used to confirm the successful synthesis and structural integrity of the PDIEH and PDIIN derivatives.

The successful imidization of the dianhydride precursor was confirmed using FT-IR spectroscopy, as illustrated in Figure 2a. Both compounds showed characteristic imide C=O stretching bands near ∼1690 cm^–1^ and ∼1640 cm^–1^, and C–N stretching vibrations near ∼1375 cm^–1^. The absence of anhydride peaks (∼1770 cm^–1^) confirmed complete ring closure.

**Figure 2:**
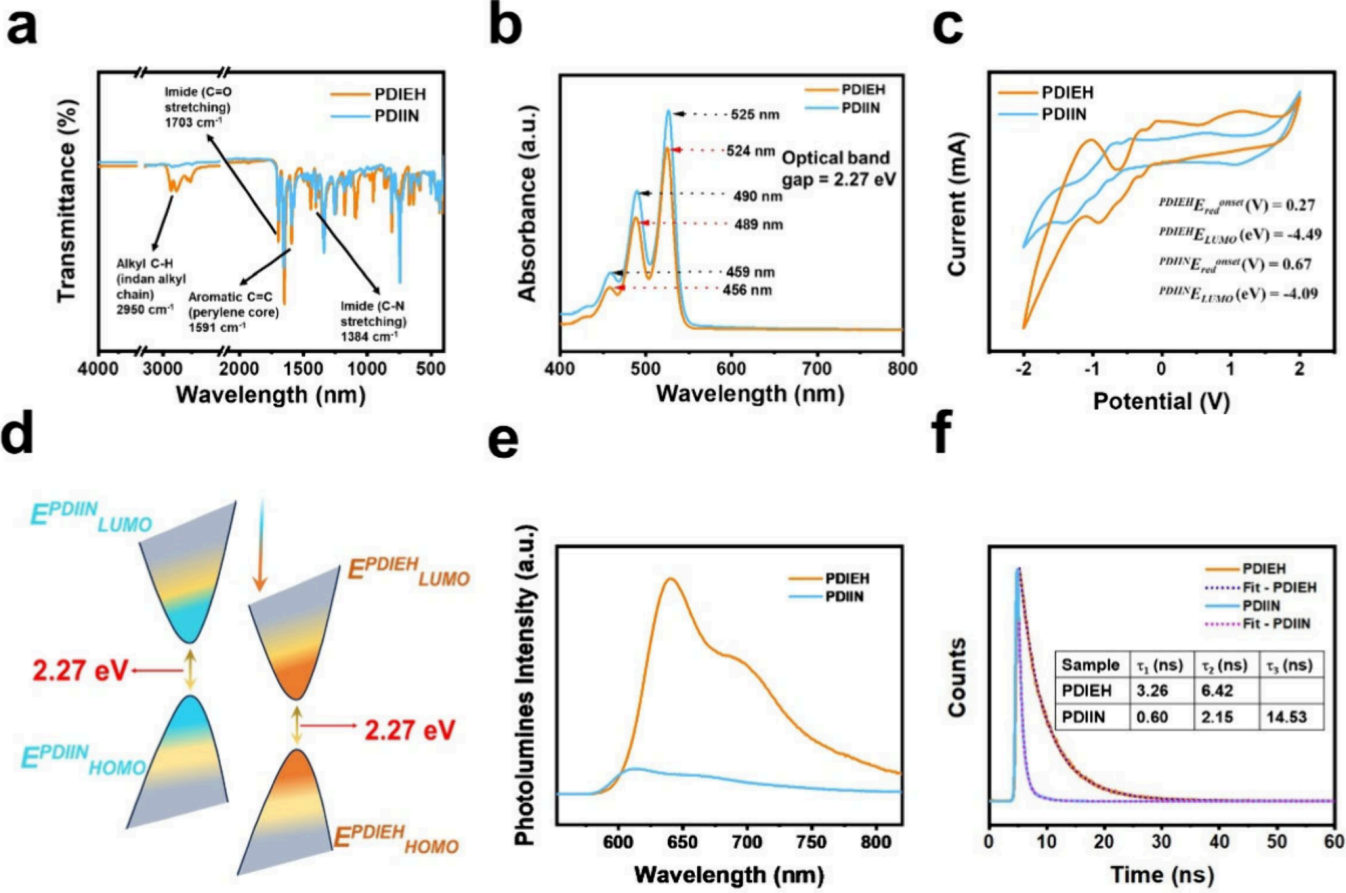
FTIR, UV–vis, cyclic voltammetry (CV), band alignment, and emission spectroscopy analysis of PDIEH and PDIIN. (a) FTIR spectra confirm characteristic vibrations of the perylene core and alkyl chains. (b) UV–vis spectra show strong visible absorption with distinct vibronic features. (c) CV curves reveal deeper LUMO levels for PDIEH, indicating more profound electron affinity and favorable energy alignment for OPV applications. (d) Energy level diagram comparing the HOMO and LUMO levels of PDIIN and PDIEH non-fullerene acceptors. Both materials exhibit an identical optical band gap of 2.27 eV. (e) Photoluminescence spectra comparing PDIEH and PDIIN emission intensity. (f) Time-resolved photoluminescence decay profiles and (inset) decay parameters illustrating exciton lifetimes for PDIEH and PDIIN.

### 3.2 Optical and Electrochemical Properties

Following the successful synthesis of the PDI-based NFAs, a thorough investigation was conducted into their optical and electrochemical properties to assess their suitability for application in organic solar cells. The optical properties were first characterized using UV–vis spectroscopy, which revealed the electronic transitions within the synthesized materials, as illustrated in Figure 2b. UV–vis absorption spectroscopy revealed that both PDIIN and PDIEH display three distinct absorption peaks linked to vibronic transitions inherent to their π–π* electronic transitions. The absorption profile for PDIIN is slightly red-shifted compared to PDIEH, with primary absorption peaks observed at 525, 490, and 459 nm for PDIIN versus 524, 489, and 456 nm for PDIEH. This red shift in PDIIN is likely due to the extended conjugation provided by the naphthalene groups, allowing it to absorb light at slightly longer wavelengths. A comparison of theoretical and experimental absorption profiles shows dominant peaks for both PDIEH and PDIIN, corresponding to distinct vibronic transitions. PDIIN exhibits peaks at 525, 490, and 459 nm, whereas PDIEH displays peaks at 524, 489, and 456 nm. The red shift in PDIIN indicates stronger conjugation from the indanyl side chains, as predicted by our DFT calculations. Additionally, the excited-state energies (S_1_ = 2.30 eV for PDIIN and 2.29 eV for PDIEH) are consistent with the measured optical gaps of 2.27 eV. These findings validate our computational approach and connect molecular design to the optical properties observed in thin films, demonstrating the predictive power of quantum-chemical simulations for device optimization.

The HOMO and LUMO levels were determined by examining their redox characteristics and energy levels using cyclic voltammetry (CV),^35^ as shown in Figure 2c, in order to gain a better understanding of their electrochemical behaviors. This investigation employed a three-electrode system consisting of a platinum wire counter electrode, a platinum disc working electrode, and an Ag/AgCl reference electrode. CV results for PDIEH and PDIIN show three distinct reduction peaks, highlighting their strong electron-accepting properties. The energy levels of the LUMO and HOMO were determined using the onset reduction potential using *E*_LUMO_ = −(*E*_onset(red)_ – 4.76 eV). The first reduction peak, occurring at 0.27 V for PDIEH and 0.67 V for PDIIN, indicates the formation of a stable radical anion and suggests that PDIEH has a higher electron affinity. The second peak reflects the formation of a dianion, while the third peak corresponds to a trianion, demonstrating the ability of these molecules to accept multiple electrons. This multistep reduction behavior highlights the strong electron-deficient nature of the perylene diimide (PDI) framework, influenced by the substituents on each compound. The DFT findings are consistent with the CV data (Table 1), as the calculated energy levels corroborate the experimentally observed trends in reduction potentials. The differences in the HOMO and LUMO positions for NFAs from the distinct approaches of CV and DFT. CV captures redox potentials in solution, reflecting both molecular properties and environmental effects, while DFT calculates energy levels for isolated molecules in the gas phase, neglecting intermolecular interactions and solid-state effects. Consequently, DFT often fails to account for shifts due to molecular packing and polarization in operational organic solar cells, resulting in significant discrepancies between energy levels obtained from CV and DFT and the actual energy landscape relevant for device performance.^36^ Moreover, the multielectron reduction behavior observed in CV can be attributed to the electronic stability of the PDI core and the minimal structural distortion upon electron addition, as indicated by the DFT-optimized geometries. A comparative energy level analysis shows that the two NFAs, PDIIN and PDIEH, exhibit identical optical band gaps of 2.27 eV, as indicated by the red arrows marking the energy difference between their respective HOMO and LUMO levels, as shown in Figure 2d. Despite sharing the same energy gap, these materials differ in their side chains: PDIIN contains a rigid, while PDIEH incorporates a flexible moiety. These structural variations, although not influencing the optical gap directly, can significantly impact intermolecular interactions and charge transport properties. This subtle tuning of energy levels through side-chain engineering offers a powerful strategy to optimize photovoltaic performance, allowing for electronic improvements without compromising the fundamental optical properties. The understanding of the materials’ optoelectronic behavior, energy level alignment with common donor polymers (P3HT and PTB7), and their anticipated performance in bulk heterojunction (BHJ) solar cells was provided by these experimental results. The band alignment diagrams for PDIIN and PDIEH (Figure S2) are emphasized as compatible with widely used donor materials such as P3HT (HOMO = −5.20 eV, LUMO = −3.10 eV) and PTB7 (HOMO = −5.20 eV, LUMO = −3.5 eV). It is noted that the LUMO levels of PDIIN (−4.09 eV) and PDIEH (−4.49 eV) are significantly deeper than those of the donor materials, creating a favorable energy offset. This offset is driven efficiently by electron transfer from the donor to the acceptor material, which is critical for achieving high charge separation efficiency and minimizing energy losses during the electron transfer process. The lower LUMO level of PDIEH (−4.49 eV) compared to PDIIN is regarded as particularly significant. Furthermore, it is understood that a deeper LUMO level reduces the electron affinity mismatch between the acceptor material and the electrode, facilitating deeper electron injection into the cathode. Additionally, it is noted that the lower LUMO level in PDIEH contributes to its ability to capture lower-energy photons from the donor material during photoexcitation, thereby extending the operational spectral range of the organic solar cell (OSC). This ensures that more photons are utilized effectively, broadening the light-harvesting capabilities of the active layer. The overall deep energy levels of PDIEH are considered particularly advantageous for improving the thermal stability of the device, as deeper energy levels reduce the likelihood of oxidative degradation.

**Table 1:** Calculated LUMO and HOMO Energy Levels of the PDI Derivative Using Empirical Formulas and Density Functional Theory (DFT) Calculations, Respectively, Alongside the Corresponding Bandgap Values Determined from Optical Absorption Onsets

compound	*E*_red_^onset^ (V)	LUMO (eV)	*E*_g_ (eV)	HOMO (eV)
PDIIN	0.67	–4.09	2.27	–6.36
PDIIN (DFT)	–	–3.45	2.52	–5.97
PDIEH	0.27	–4.49	2.27	–6.76
PDIEH (DFT)	–	–3.45	2.53	–5.98

To investigate the influence of side-chain design on molecular packing, scanning electron microscopy (SEM) and grazing incidence X-ray diffraction (GIXRD) characterization of all blend films were performed (Figures S4 and S5, respectively). In Figure S4, SEM images reveal that PDIEH-based blends (P3HT:PDIEH, PTB7:PDIEH) produce much smoother and more homogeneous film surfaces, lacking substantial aggregates or phase boundaries. In comparison, PDIIN-based blends (P3HT:PDIIN, PTB7:PDIIN) show higher surface roughness and pronounced domain features, indicative of increased phase separation. Figure S5 presents the corresponding GIXRD profiles, all blends display an intense diffraction peak near 2θ = 4–5°. This peak is clearest and most intense for PDIEH blends, pointing to enhanced molecular order, while the same feature is weaker and broader for PDIIN blends, confirming their greater disorder. No strong π–π stacking peaks are observed at higher angles, suggesting that long-range stacking predominates over vertical crystalline coherence in these systems. These SEM and GIXRD results clearly demonstrate that the flexible PDIEH side chain promotes nanoscale phase uniformity and crystallinity, while the rigid PDIIN side chain results in less-ordered, phase-separated structures. Additionally, the photoluminescence (PL) studies were conducted on the thin films deposited onto a quartz substrate to investigate exciton dynamics and charge recombination rates. The PL data revealed that PDIEH exhibits an intense PL peak around 650 nm, starkly contrasting the significantly lower intensity observed in PDIIN, as shown in Figure 2e. This higher PL intensity for PDIEH suggests better radiative recombination efficiency. In contrast, the reduced intensity in PDIIN may indicate a higher prevalence of nonradiative recombination pathways, potentially stemming from structural differences, defects, or aggregation effects. Such observations are closely related to molecular design and the extent of molecule interactions, which can lead to differing excitonic behaviors. Time-resolved photoluminescence (TRPL) data further elucidate this, showing that PDIIN has three decay constants: τ_1_ = 0.60 ns, τ_2_ = 2.15 ns, and τ_3_ = 14.53 ns, as shown in Figure 2f. The shortest decay component (τ_1_) is associated with nonradiative decay processes, and the intermediate and long components imply multiple relaxation pathways, highlighting the dominance of nonradiative decay as a contributor to the reduced PL intensity. Conversely, PDIEH has two decay constants, τ_1_ = 3.26 ns and τ_2_ = 6.42 ns, showcasing longer average decay times than PDIIN, indicative of more efficient radiative recombination and fewer nonradiative pathways. The intense PL exhibited by PDIEH aligns well with the longer TRPL decay times, signifying efficient exciton lifetimes. In contrast, PDIIN’s weak PL and shorter TRPL components reveal the prevalence of nonradiative recombination that defects and molecular packing could influence. PDIEH demonstrates superior radiative recombination capabilities, making it a more suitable candidate for optoelectronic applications. At the same time, PDIIN’s efficiency can potentially be improved by addressing its nonradiative losses through processing or molecular engineering. The findings from both PL and TRPL results establish a robust basis for pursuing further ultrafast studies to dissect excitonic and charge dynamics.

### 3.3 Charge Transfer and Ultrafast Transient Absorption Spectroscopy

Ultrafast transient absorbance spectroscopy (UTAS), which builds on the understanding from PL and TRPL, is a potent method that allows us to study the fast dynamics of charge transfer and carrier interactions on a nanosecond to femtosecond time scale. UTAS was employed to unravel the excited-state dynamics and charge-transfer processes in both pristine materials and their polymer blends. For every UTAS experiment, the kinetic fitting of transient absorption traces produces several well-defined parameters: (i) the characteristic lifetime constants (τ_1_, τ_2_, τ_3_, τ_4_) correspond to physical processes such as ultrafast charge transfer, polaron separation and stabilization, charge trapping, and eventual recombination; (ii) kinetic components refer to amplitude coefficients (*A*_1_–*A*_4_), which quantify the contribution of each relaxation process to the overall photophysical response; (iii) signal assignments include ground-state bleach (GSB, negative signal), photoinduced absorption (PIA, positive signal from excited-state absorption). For instance, GSB identifies the depopulation of the ground-state due to photoexcitation, and PIA marks the creation of excited-state carriers (Table S4). UTAS measurements on pure thin films of PDIIN and PDIEH revealed notable differences in their excited-state behavior attributed to their molecular structures, as shown in Figure S4, S5. Upon excitation at 500 nm, the signal is probed in the visible range (510 to 800 nm), and both materials exhibited intense GSB signals at 538 and 532 nm, respectively, along with PIA at 770 and 699 nm, respectively. These findings indicate effective excitation of the π-conjugated system. PDIIN’s PIA signal peaked at ∼770 nm and exhibited long-lived states with a slowest decay lifetime of τ_3_ ≈ 3020 ps and an average of ∼2422 ps, suggesting stabilized charge-separated states and reduced recombination due to its rigid, planar indanyl groups. In contrast, PDIEH displayed a PIA band at ∼699 nm that decayed more quickly, indicating more transient excited states with a slowest decay lifetime of t_3_ ∼ 4350 ps, and an average lifetime of ∼3120 ps, and the GSB signal at ∼532 nm had a longer average lifetime of more than 6 ns, pointing to higher stabilization of charge-separation, influenced by its flexible ethylhexyl chains. These findings highlight how side-chain structure affects charge dynamics in NFAs. Tables S5 and S6 summarize the kinetic fitting parameters from the transient absorption data for both materials.

The excited-state dynamics and charge-transfer mechanisms in polymer blends of P3HT and PTB7 with NFAs, PDIEH, and PDIIN were investigated using UTAS in order to gain a better understanding of their exciton dynamics, as shown in Figure 3. Both blend systems exhibited strong GSB signals at 550 and 560 nm aligned with P3HT and NFAs blends, suggesting efficient exciton dissociation and rapid charge transfer at the interfaces. Significant PIA signal at 660 and 670 nm, features indicated effective polaron formation and stabilization, as shown in Figure 3b,e. Transient kinetic profile (TKP) analyses using multiexponential fitting revealed multiple kinetic components, including ultrafast charge transfer (τ_1_ ∼ hundreds of femtoseconds), charge separation and polaron stabilization (τ_2_ ∼ tens of picoseconds), intermediate charge trapping (τ_3_ ∼ tens to hundreds of picoseconds), and slower recombination processes (τ_4_ ∼ a few nanoseconds), as shown in Figure 3c,f. Table 2 lists the kinetic fitting parameters from the transient absorption data for P3HT blends. Comparative analysis between P3HT:PDIEH and P3HT:PDIIN blends elucidated subtle yet significant kinetic differences. The two blends exhibited comparable ultrafast charge transfer and polaron separation kinetics (τ_1_ ≈ 0.52 ps, *A*_1_ ≈ 77.1% and τ_2_ ≈ 7.90 ps, *A*_2_ ≈ 12.6% for PDIIN; τ_1_ ≈ 0.566 ps, *A*_1_ ≈ 75.1% and τ_2_ ≈ 7.27 ps, *A*_2_ ≈ 12.0% for PDIEH). Notably, substantial differences were observed in the intermediate and longer-lived kinetic processes. Although PDIIN facilitated quicker ultrafast CT and comparable polaron separation processes, the PDIIN-based system exhibited more rapid intermediate charge trapping (τ_3_ = 89.9 ps) than that of PDIEH (τ_3_ ≈ 109.00 ps), suggesting accelerated, albeit potentially less energetically favorable, trapping pathways. In contrast, the PDIEH blend demonstrated significantly prolonged carrier recombination lifetimes (τ_4_ ≈ 5.35 ns, *A*_4_ = 4.5%) relative to PDIIN (τ_4_ ≈ 4.26 ns, *A*_4_ = 3.0%), reflecting suppressed nonradiative losses and enhanced carrier persistence. These disparities in kinetic lifetimes emphasized the impact of structural modifications of NFAs on electronic coupling at the donor–acceptor interface, significantly influencing exciton dissociation efficiency, charge trapping tendencies, and recombination losses.

**Table 2:** Kinetic Fitting Parameters for P3HT Blends at Ground State Bleaching (GSB)^a^

parameter	P3HT:PDIEH	P3HT:PDIIN
GSB: τ_1_ (ps)	0.57 (75.1%)	0.52 (77.1%)
GSB: τ_2_ (ps)	7.27 (12.0%)	7.90 (12.6%)
GSB: τ_3_ (ps)	109.00 (8.3%)	89.90 (7.2%)
GSB: τ_4_ (ns)	5.35 (4.5%)	4.26 (3.0%)

a τ_1_, τ_2_, τ_3_, and τ_4_ represent the decay time constants associated with charge transfer (CT), separated polarons (SP) (dissociated BPP), trapped BPP, and the recombination of charge carriers, respectively. The coefficients *A*_1_, *A*_2_, *A*_3_, and *A*_4_ represent the proportions of carriers undergoing relaxation via each process.

**Figure 3:**
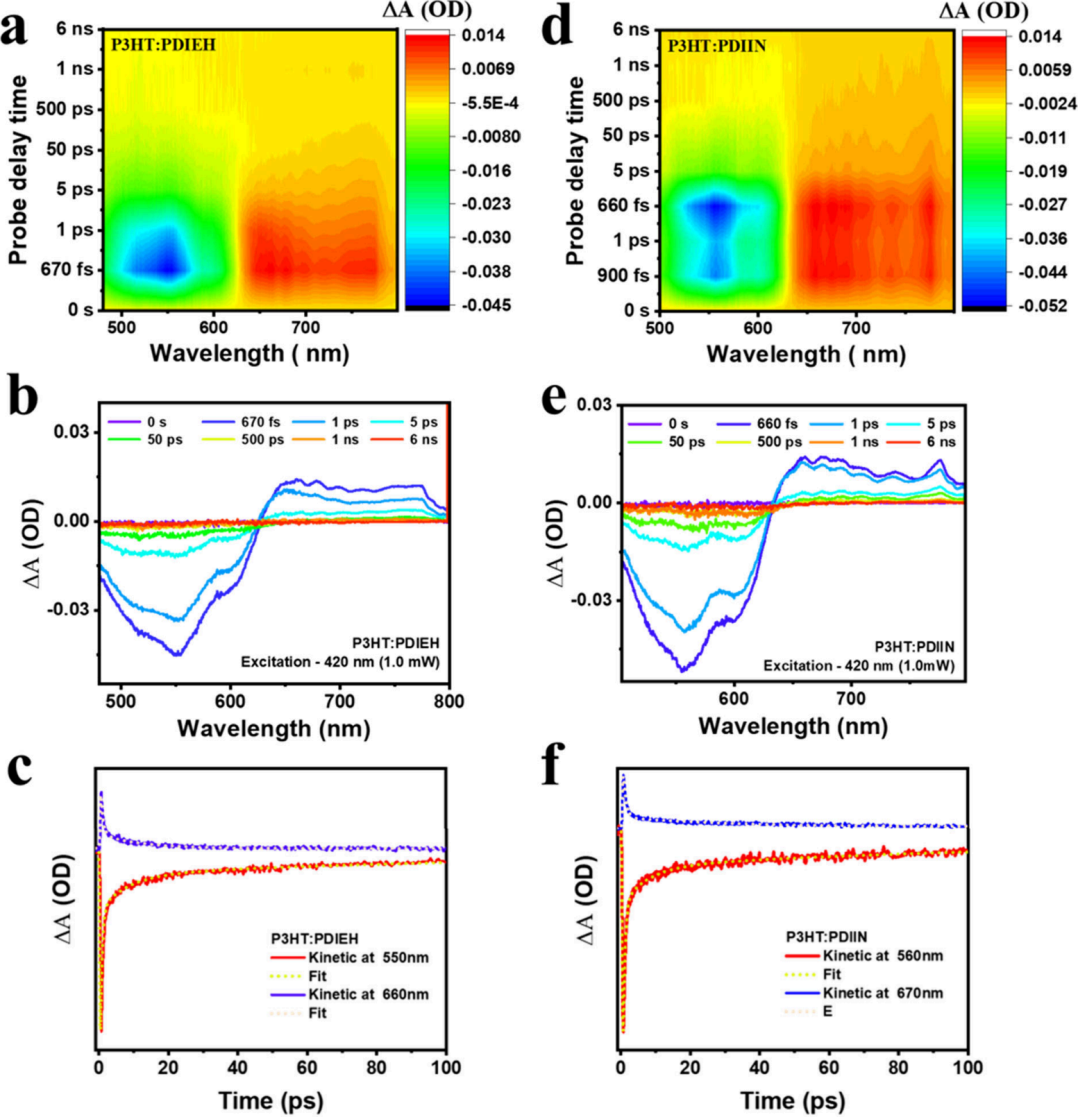
UTAS analysis of P3HT:PDIEH (left) and P3HT:PDIIN (right) blends under 420 nm excitation. (a, d) 2D-color contour plots showing differential optical density (Δ*A*) as a function of wavelength and delay time, revealing distinct photoinduced dynamics. (b, e) Evolution-associated spectra (EAS) from global fitting, highlighting key processes such as exciton dissociation, charge separation, and recombination with corresponding lifetimes. (c, f) Kinetic traces at selected probe wavelengths with multiexponential global fits, demonstrating trapping dynamics were slower in the PDIEH blend (τ_3_ ≈ 109 ps, *A*_3_ ≈ 8.35%) compared to the PDIIN blend (τ_3_ ≈ 89.90 ps, *A*_3_ ≈ 12.60%). Furthermore, recombination dynamics were significantly delayed in the PDIEH blend (τ_4_ ≈ 5.35 ns, *A*_4_ ≈ 4.55%) relative to the PDIIN blend (τ_4_ ≈ 4.36 ns, *A*_4_ ≈ 3.03%). Faster exciton quenching and more sustained charge-separated states in the PDIEH-based blend than in PDIIN.

The dynamics and charge-transfer mechanisms in polymer blends of PTB7 with NFAs, PDIEH, and PDIIN were studied using UTAS to understand their exciton dynamics, as shown in Figure 4. The TSPs reveal distinct photoinduced phenomena, including pronounced GSB at 627 and 640 nm, as well as PIA at 764 and 770 nm. These findings indicate efficient exciton dissociation and the formation of charge-separated states, primarily polarons. The blends of PTB7:PDIEH and PTB7:PDIIN exhibited strong GSB signatures, confirming effective charge separation at the donor–acceptor interface, and PIA features indicated the stabilization of charge carriers, highlighting the significance of long-lived polarons for photovoltaic performance, as shown in Figure 4b,e. Table 3 presents the kinetics from the transient absorption data for the PTB7 blends.

**Table 3:** Kinetic fitting parameters for PTB7 blends at Ground State Bleaching (GSB)^a^

parameter	PTB7:PDIIN	PTB7:PDIEH
GSB: τ_1_ (ps)	0.47 (72.9%)	0.41 (72.3%)
GSB: τ_2_ (ps)	5.04 (17.9%)	5.63 (16.1%)
GSB: τ_3_ (ps)	55.90 (6.8%)	154.00 (6.8%)
GSB: τ_4_ (ns)	3.89 (2.3%)	6.59 (4.8%)

a τ_1_, τ_2_, τ_3_, and τ_4_ represent the decay time constants associated with charge transfer (CT), separated polarons (SP) (dissociated BPP), trapped BPP, and the recombination of charge carriers, respectively. The coefficients *A*_1_, *A*_2_, *A*_3_, and *A*_4_ represent the proportions of carriers undergoing relaxation via each process.

**Figure 4:**
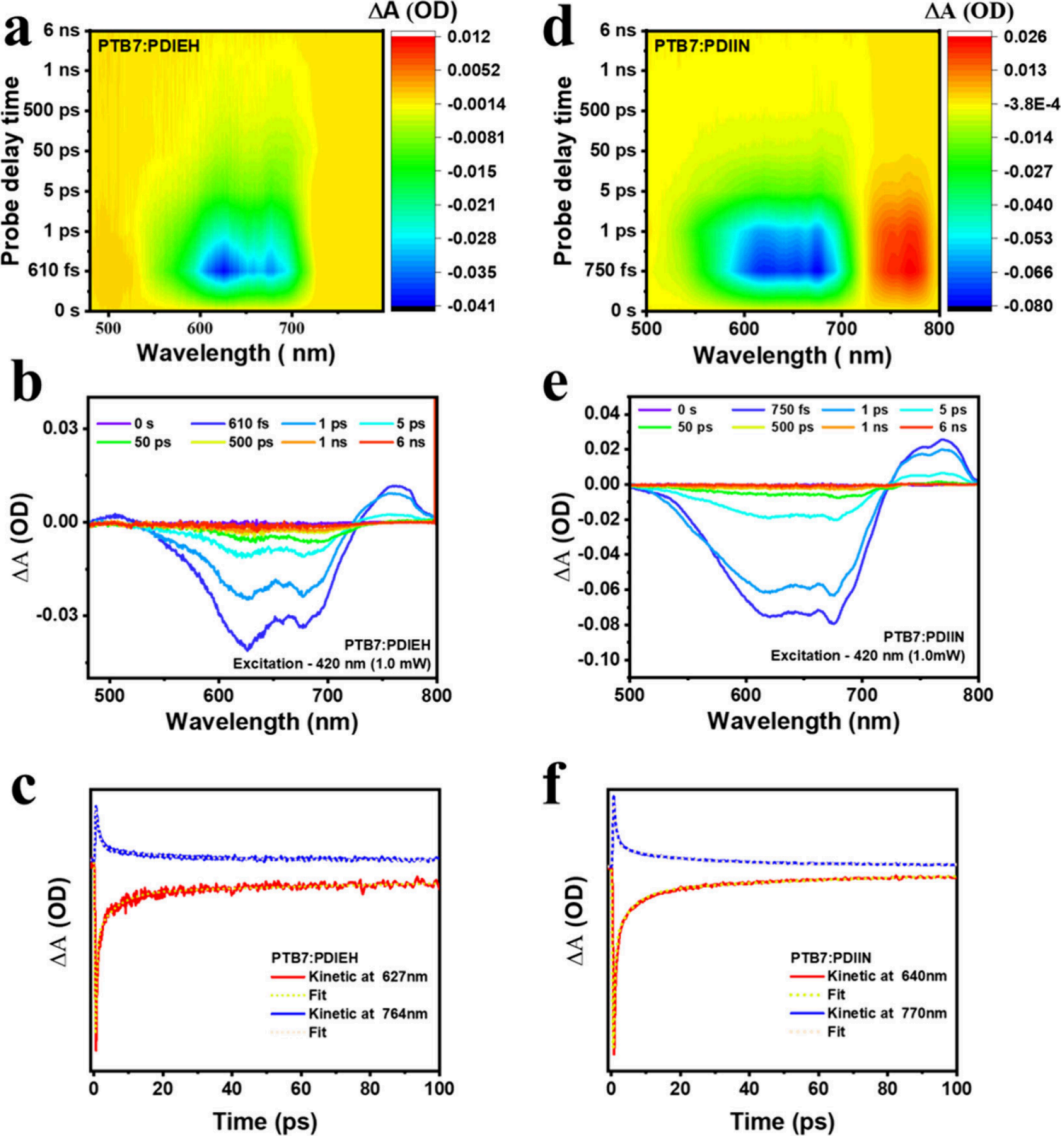
UTAS analysis of PTB7:PDIEH (left) and PTB7:PDIIN (right) blends under 420 nm excitation. (a, d) 2D-color contour plots showing differential optical density (Δ*A*) as a function of wavelength and delay time, revealing distinct photoinduced dynamics. (b, e) Evolution-associated spectra (EAS) from global fitting, highlighting key processes such as exciton dissociation, charge separation, and recombination with corresponding lifetimes. (c, f) Kinetic traces at selected probe wavelengths with multiexponential global fits. The PDIEH blend exhibits longer-lived charge-separated states and more prominent excited-state features than PDIIN, indicating more efficient charge stabilization and reduced recombination losses.

Comprehensive TKP analyses, utilizing multiexponential fitting models, resolved four distinct dynamical regimes: ultrafast charge transfer (CT, τ_1_ ≈ 0.47 ps for PDIIN and 0.41 ps for PDIEH), charge separation from BPP to SP and polaron stabilization (τ_2_ ≈ 5.63 ps for PDIEH and 5.04 ps for PDIIN), intermediate carrier trapping (τ_3_ ≈ 154.00 ps for PDIEH and 55.90 ps for PDIIN), and delayed carrier recombination processes (τ_4_ ≈ 6.59 ns for PDIEH and 3.89 ns for PDIIN). Comparative kinetic assessments revealed critical disparities in photophysical behavior. Although both blends facilitated comparable ultrafast CT and polaron separation processes, the PDIIN-based system exhibited more rapid intermediate charge trapping (τ_3_ ≈ 55.9 ps) yet with identical amplitude contribution (6.8%) to that of PDIEH (τ_3_ ≈ 154 ps), suggesting accelerated, albeit potentially less energetically favorable, trapping pathways. In contrast, the PDIEH blend demonstrated significantly prolonged carrier recombination lifetimes (τ_4_ ≈ 6.59 ns, *A*_4_ = 4.7%) relative to PDIIN (τ_4_ ≈ 3.89 ns, *A*_4_ = 2.3%), reflecting suppressed nonradiative losses and enhanced carrier persistence. These disparities in kinetic lifetimes and amplitude distributions emphasize the profound influence of acceptor molecular architecture on interfacial electronic coupling, exciton dissociation efficiency, charge-carrier delocalization, and recombination dynamics. PDIEH is characterized by superior suppression of recombination events and extended polaron lifetimes, whereas PDIIN facilitates rapid charge extraction and efficient early time carrier dynamics. The ultrafast spectroscopic investigation reveals that although PDIIN and PDIEH serve as efficacious non-fullerene acceptors in PTB7-based systems, PDIEH exhibits superior photophysical traits, particularly regarding carrier longevity and recombination suppression. These attributes underscore its potential as a more effective molecular acceptor in high-performance organic photovoltaic devices.

The global fitting for the blends of P3HT and PTB7 was done using Surface Explorer, and the comparative global kinetic analysis of decay-associated difference spectra, as shown in Figure 5 for P3HT and PTB7 donor blends with PDIEH and PDIIN non-fullerene acceptors, reveals significant differences in their photoinduced charge dynamics, highlighting the critical influence of molecular structure on exciton dissociation and charge carrier behavior. In the ultrafast temporal regime (τ_1_), PDIEH-based blends—P3HT:PDIEH (∼569.4 fs) and PTB7:PDIEH (∼577.5 fs)—exhibit faster decay constants relative to PDIIN-based systems—P3HT:PDIIN (∼661.2 fs) and PTB7:PDIIN (∼612.6 fs). These subpicosecond time scales indicate highly efficient exciton quenching at the donor–acceptor interface, with PDIEH facilitating more rapid electron transfer, likely due to favorable energy level alignment and stronger electronic coupling. In the subsequent charge separation phase (τ_2_), PDIEH and PDIIN both support efficient polaron formation; however, PTB7:PDIIN exhibits the shortest lifetime (∼8.2 ps), suggesting an accelerated charge carrier diffusion toward the heterojunction interface, potentially driven by optimized donor–acceptor morphology or enhanced dielectric screening.

**Figure 5:**
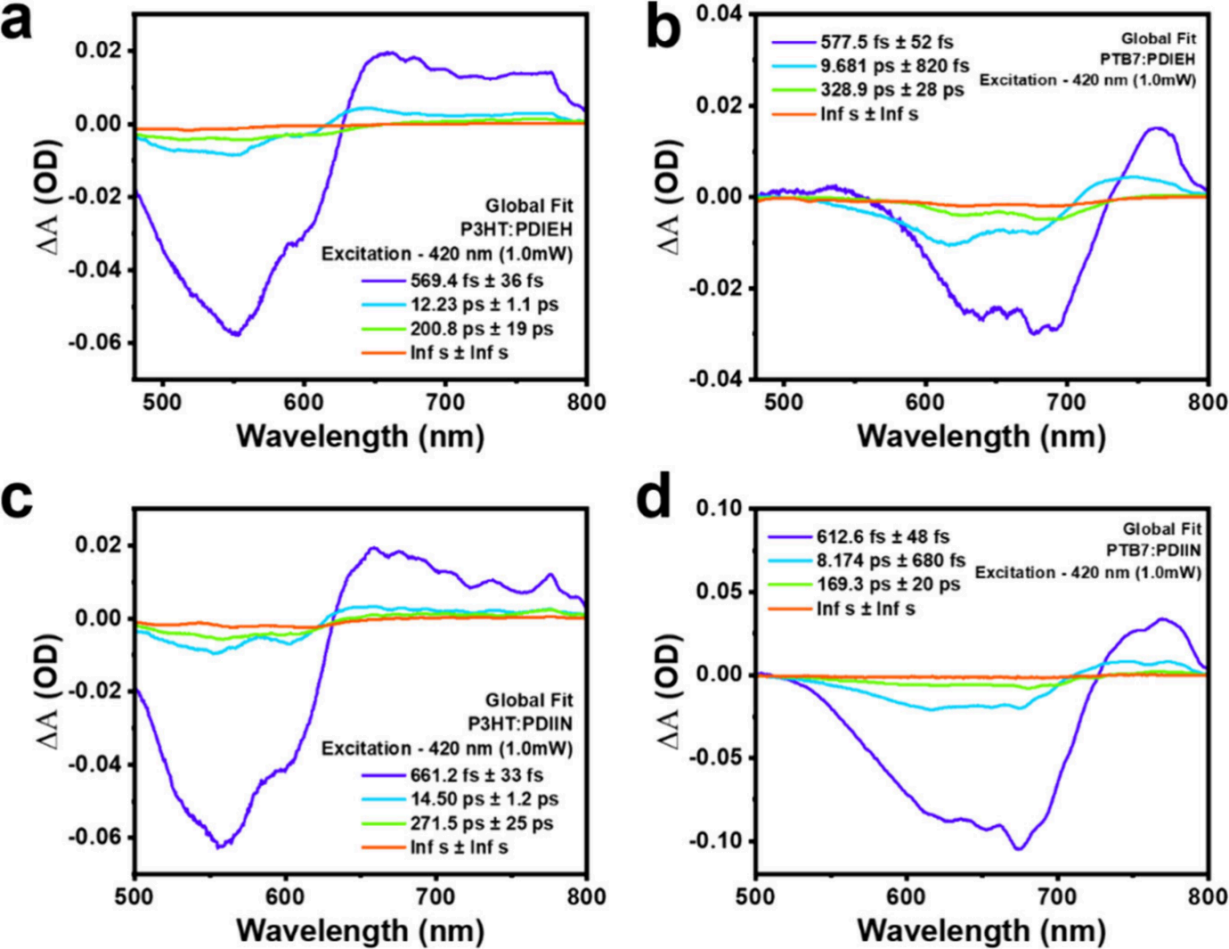
Global fitting of transient absorption spectra for donor–acceptor blends under 420 nm excitation. (a) P3HT:PDIEH, (b) PTB7:PDIEH, (c) P3HT:PDIIN, and (d) PTB7:PDIIN. The fitted lifetimes represent exciton dissociation, charge separation, trapping, and recombination processes. PDIEH blends show faster charge transfer and longer-lived charge-separated states.

The longer-lived decay component (τ_3_) associated with the lifetime of separated charges or trapped bound polaron pairs reveals a distinct advantage for PDIEH systems. The PTB7:PDIEH blend shows a significantly extended lifetime (∼328.9 ps) compared to PTB7:PDIIN (∼169.3 ps), implying reduced nongeminate recombination and superior stabilization of charge-separated states. This may be attributed to the planar molecular backbone and side-chain induced packing behavior of PDIEH, which could facilitate more favorable phase segregation and percolation pathways for charge transport. Moreover, the transient absorption signal intensity (Δ*A*), particularly the GSB and PIA features, is more pronounced in PDIEH blends, further supporting the notion of enhanced photoinduced charge generation and accumulation. Taken together, these observations establish PDIEH as a more efficient acceptor material compared to PDIIN, especially in blends with PTB7, due to its ability to simultaneously promote ultrafast charge transfer and maintain long-lived charge-separated states, both of which are essential for achieving high power conversion efficiency in organic photovoltaic devices.

## 4 Conclusion

This study introduces a sophisticated approach to side-chain engineering that allows for the precise modulation of absolute frontier orbital energies in non-fullerene acceptors (NFAs), while keeping their optical band gaps unchanged. By examining two NFAs, PDIEH, which features flexible and electronically inert alkyl chains, and PDIIN, which incorporates rigid, partially conjugated indanyl groups, we demonstrate how different side-chain substituents can finely tune the HOMO and LUMO energy levels via inductive and conjugative effects. Our findings are anchored in detailed electronic structure and excited state simulations, supported by cyclic voltammetry and UV–vis spectroscopy. These analyses show that the flexible side chains of PDIEH contribute to a stabilization of the electronic structure through inductive effects, resulting in deeper energy states. In contrast, the rigid side chains in PDIIN promote a slight delocalization and destabilization of orbitals. Notably, despite both NFAs exhibiting similar optical gaps of approximately 2.27 eV, the differential absolute energy levels profoundly affect exciton dynamics. This is particularly illustrated through ultrafast transient absorption spectroscopy (UTAS), which reveals that PDIEH facilitates longer-lived charge-separated states compared to PDIIN. These nuanced energy-level modifications are crucial for influencing charge separation and recombination processes, thereby offering a predictive and intuitive framework for the rational design of next-generation organic semiconductors.

## Supplementary Material



## Notes

2D contour plot, UTAS spectra, kinetic fitting spectra for PDIEH and PDIIN, and DFT coordinates (PDF)
