# Supplementary material for: Probing Side-Chain Engineering for Modulating Exciton Dynamics in Non‑fullerene Acceptors
Source: ACS Omega. 2025 Nov 18;11(1):1125–35. doi: 10.1021/acsomega.5c08337 (PMC12809543; doi:10.1021/acsomega.5c08337)
Supplement: Supplementary file 1 [file ao5c08337_si_001.pdf]

Supporting Information

Probing Side-Chain Engineering for Modulating Exciton Dynamics in Non-Fullerene Acceptors

*Sanyam Jain[1,2], M Sridevi[1,2], Tanushree Majhi[1,2], Narendra Pratap Tripathi[3], Saurabh Kumar Saini[4], Anita Kumari[3], Sanchita Sengupta[3], Rajiv K. Singh[1,2]**

[1]Photovoltaic Metrology Section, Advanced Materials & Device Metrology Division, CSIR-National Physical Laboratory, Dr K. S. Krishnan Marg, New Delhi-110012, India

[2]Academy of Scientific and Innovative Research (AcSIR), Ghaziabad-201002, India

[3]Department of Chemical Sciences, Indian Institute of Science Education and Research (IISER) Mohali, Knowledge City, Sector 81, Punjab-140306, India.

[4]Department of Physics, Indian Institute of Technology Roorkee, Roorkee, Uttarakhand 247667, India.

*Email - rajivsingh@nplindia.res.in

Table of Contents

1. Density Functional Theory **S3**

2. Table S1: FMO energy levels of compounds **S4**

3. Table S2: Singlet and triplet energies of PDIIN and PDIEH **S5**

4. Table S3: Results of the first three frequencies and molecular symmetries **S6**

5. DFT Coordinates **S7**

6. Structural Characterization via NMR Spectroscopy **S10**

7. Figure S1: ^{1}H-NMR and ^{13}C-NMR spectra for PDIEH and PDIIN **S11**

8. Figure S2: Energy level alignment **S12**

9. Figure S3: UV-Vis Spectra **S13**

10. Figure S4: SEM Images **S14**

11. Figure S5: GIXRD Spectra **S15**

10. Sample preparation **S16**

11. UTAS calculations **S17**

12. Table S4: Summary of UTAS parameters **S18**

13. Figure S6: UTAS spectra (PDIIN) ` **S19**

14. Table: S5 Kinetic Fitting Parameters (PDIIN) **S20**

15. Figure S7: UTAS spectra (PDIEH) **S21**

16. Table: S6 Kinetic Fitting Parameters (PDIEH) **S22**

17. Reference **S23**

DENSITY FUNCTIONAL THEORY

Density functional theory (DFT) calculations were performed on PDIIN and PDIEH in the ground state using the Gaussian16 program suite. [S1] The studied molecules were optimized using the global hybrid B3LYP functional and 6–31G (d, p) basis in the gas phase. The frontier molecular orbitals (FMO) energy levels were obtained from geometry optimization of neutral ground states. Furthermore, time-dependent DFT calculations were also performed using the B3LYP functional and a 6-31G (d, p) basis set using the CPCM model and chloroform solvent.

Table S1. FMO energy levels of compounds of PDIIN and PDIEH calculated by B3LYP/6-31G (d, p).

**Compound**	**HOMO–1 (eV)**	**HOMO (eV)**	**LUMO (eV)**	**LUMO +1 (eV)**	**ΔE (Bandgap)**
**PDIIN**	−6.26	−5.97	−3.45	1.86	2.52
**PDIEH**	−7.23	−5.98	−3.45	−1.86	2.53

Table S2. Singlet and triplet energies of PDIIN and PDIEH calculated by time-dependent DFT using B3LYP/6-31G (d, p).

Compound	S1(eV)	S2 (eV)	T1 (eV)	T2 (eV)	T3 (eV)	Δ E_{ST} (eV) (S1-T1)
**PDIIN**	2.29	2.63	1.24	2.60	2.61	1.05
**PDIEH**	2.30	3.30	1.24	2.67	2.77	1.06

Table S3: Results of the first three frequencies and molecular symmetries calculated from geometry optimization of PDIIN and PDIEH

Compound	First three frequencies	Symmetry
**PDIIN**	12.34<br>12.71<br>23.47	C1
**PDIEH**	10.37<br>12.00<br>17.93	C1

Coordinates for the optimized structure of PDIIN

C	-1.43042	0.55513	0.35746
C	-0.73500	-0.64340	0.71471
C	0.73525	-0.64336	0.71465
C	1.43056	0.55523	0.35739
C	0.73519	1.75332	-0.00108
C	-0.73516	1.75327	-0.00105
C	2.85943	0.55562	0.35850
C	3.57411	1.72457	0.00685
C	2.88169	2.87255	-0.33856
C	1.48290	2.88303	-0.33971
C	1.48323	-1.77320	1.05209
C	2.88209	-1.76214	1.05214
C	3.57401	-0.61317	0.71019
C	-2.85929	0.55540	0.35866
C	-3.57377	-0.61345	0.71032
C	-2.88175	-1.76234	1.05230
C	-1.48289	-1.77327	1.05229
C	-1.48297	2.88292	-0.33963
C	-2.88177	2.87236	-0.33833
C	-3.57408	1.72432	0.00712
C	5.05773	1.74138	-0.00776
C	5.05788	-0.62739	0.72616
N	5.71796	0.55706	0.37026
C	-5.05770	1.74105	-0.00721
N	-5.71786	0.55663	0.37068
C	-5.05767	-0.62784	0.72612
O	5.68137	2.74331	-0.33609
O	5.68639	-1.62800	1.04449
O	-5.68147	2.74299	-0.33528
O	-5.68602	-1.62873	1.04391
C	7.21030	0.57310	0.34529
C	-7.21030	0.57285	0.34586
C	7.89924	0.13975	1.67432
C	8.64474	-1.19034	1.38917
C	8.64371	-1.30324	-0.11791
C	7.81130	-0.34411	-0.70170
C	9.32393	-2.21629	-0.92381
C	9.15996	-2.15927	-2.31001
C	8.32494	-1.19784	-2.88900
C	7.64583	-0.28032	-2.08450
C	-7.81157	-0.34377	-0.70150
C	-8.64345	-1.30352	-0.11796
C	-8.64398	-1.19149	1.38919
C	-7.89909	0.13884	1.67470
C	-7.64692	-0.27892	-2.08435
C	-8.32619	-1.19608	-2.88914
C	-9.16058	-2.15819	-2.31039
C	-9.32380	-2.21623	-0.92414
H	3.44580	3.75863	-0.60738
H	0.98419	3.80396	-0.61552
H	0.98453	-2.69486	1.32555
H	3.44686	-2.64860	1.31853
H	-3.44645	-2.64885	1.31867
H	-0.98410	-2.69483	1.32589
H	-0.98435	3.80388	-0.61550
H	-3.44596	3.75842	-0.60704
H	7.43592	1.61414	0.11771
H	-7.43571	1.61406	0.11891

H	7.18362	0.04766	2.49288
H	8.61348	0.91986	1.95577
H	8.11282	-2.04033	1.82810
H	9.65906	-1.18541	1.80372
H	9.97308	-2.96727	-0.48089
H	9.68798	-2.86562	-2.94449
H	8.21089	-1.16019	-3.96850
H	7.00450	0.47520	-2.53096
H	-8.11149	-2.04144	1.82748
H	-9.65818	-1.18724	1.80406
H	-7.18355	0.04702	2.49337
H	-8.61378	0.91855	1.95619
H	-7.00611	0.47713	-2.53064
H	-8.21277	-1.15756	-3.96868
H	-9.68873	-2.86427	-2.94506
H	-9.97248	-2.96772	-0.48140

Coordinates for the optimized structure of PDIEH

C	-1.71255	-0.13971	-0.43458
C	-1.07373	1.11826	-0.67404
C	0.36890	1.24746	-0.42068
C	1.09554	0.11215	0.05984
C	0.45632	-1.14532	0.30152
C	-0.98554	-1.27570	0.04417
C	2.49738	0.23820	0.30755
C	3.23918	-0.86797	0.78661
C	2.60081	-2.07416	1.01686
C	1.22932	-2.20704	0.77601
C	1.06021	2.44227	-0.63130
C	2.43286	2.55593	-0.38756
C	3.15520	1.47037	0.07694
C	-3.11429	-0.26580	-0.68279
C	-3.85799	0.84183	-1.15625
C	-3.21985	2.04936	-1.38138
C	-1.84792	2.18155	-1.14294
C	-1.67568	-2.47221	0.24921
C	-3.04771	-2.58680	0.00218
C	-3.77015	-1.49977	-0.45813
C	4.69333	-0.75198	1.05284
C	4.61033	1.62068	0.32593
N	5.29663	0.48432	0.78088
C	-5.22275	-1.64827	-0.71612
N	-5.91311	-0.51098	-1.15990
C	-5.31449	0.73162	-1.41778
O	5.35335	-1.68678	1.48920
O	5.19130	2.68290	0.14002
O	-5.80933	-2.71071	-0.55187
O	-5.96943	1.67904	-1.83497
C	6.74039	0.60286	1.07267
C	-7.35494	-0.67128	-1.44034
C	7.68384	0.00178	0.00969
C	7.49926	0.62554	-1.39721
C	9.13152	0.16950	0.52491
C	10.15987	-0.74072	-0.15913
C	6.48829	-0.09501	-2.29880
C	11.58902	-0.53501	0.35920
C	12.61277	-1.45611	-0.31101
C	-8.27732	-0.64279	-0.20355
C	-8.18337	0.67847	0.60061
C	-7.16041	0.67621	1.74687

C	-7.12575	1.99731	2.52521
C	-6.12169	1.99249	3.68176
C	-9.71867	-0.91655	-0.69065
C	-10.69006	-1.32724	0.42070
H	3.18592	-2.90899	1.38647
H	0.77248	-3.16959	0.96993
H	0.53671	3.31808	-0.99426
H	2.95372	3.49168	-0.55738
H	-3.80573	2.88617	-1.74505
H	-1.39193	3.14517	-1.33347
H	-1.15144	-3.34884	0.60910
H	-3.56775	-3.52421	0.16567
H	6.90554	0.09540	2.02518
H	6.93899	1.66855	1.19228
H	-7.46519	-1.63239	-1.94636
H	-7.62208	0.13175	-2.12830
H	7.47152	-1.07390	-0.04572
H	8.46655	0.62376	-1.91315
H	7.21677	1.67940	-1.28944
H	9.15803	-0.03553	1.60411
H	9.43428	1.22057	0.40907
H	10.14882	-0.57731	-1.24514
H	9.86653	-1.78906	-0.00757
H	6.77495	-1.14190	-2.45022
H	6.43568	0.38016	-3.28413
H	5.47796	-0.09083	-1.87999
H	11.60688	-0.69706	1.44553
H	11.88229	0.51232	0.20485
H	12.36578	-2.51075	-0.14437
H	13.62131	-1.28559	0.07943
H	12.64390	-1.29266	-1.39430
H	-7.98228	-1.47881	0.44410
H	-9.16578	0.90109	1.03583
H	-7.96477	1.50495	-0.08687
H	-6.15483	0.46289	1.36300
H	-7.39724	-0.14560	2.43783
H	-8.13038	2.21393	2.91365
H	-6.88589	2.81642	1.83403
H	-6.35605	1.20521	4.40737
H	-6.12128	2.94770	4.21694
H	-5.10216	1.81389	3.32102
H	-9.69775	-1.71505	-1.44388
H	-10.09883	-0.02297	-1.20538
H	-10.35823	-2.25021	0.90892
H	-11.69233	-1.50527	0.01786
H	-10.78129	-0.55808	1.194

Structural Characterization via NMR Spectroscopy

A comprehensive suite of characterization techniques was employed to confirm the successful synthesis and structural integrity of the PDIEH and PDIIN derivatives, including nuclear magnetic resonance (NMR) spectroscopy and Fourier-transform infrared (FTIR) spectroscopy.

The ^{1}H-NMR spectra of PDIEH and PDIIN provide detailed insight into the structures of these synthesized perylene derivatives, confirming successful functionalization and highlighting the distinct proton environments introduced by each synthesis route. The ^{1}H-NMR spectrum of PDIEH provides detailed insights into the molecular structure. The chemical shifts (δ) observed at various resonances indicate distinct proton environments within the molecule (refer to Figure 2(a)). Specifically, signals were detected at

^{1}H NMR of PDIEH (400 MHz, $CDCl_3$) δ (ppm): 8.59 (d, J = 8 Hz, 4 H), 8.48 (d, J = 8 Hz, 4 H), 4.19-4.08 (m, 4 H), 1.99-1.93 (m, 2 H), 1.42-1.32 (m,16 H), 0.97-0.88 (m,12 H).

^{1}H NMR of PDIIN (400 MHz, $CDCl_3$) δ (ppm): 8.59 (d, J = 4 Hz, 4 H), 8.52 (d, J = 8 Hz, 4 H), 7.34 (d, J = 8 Hz, 4 H), 7.15-7.12 (m, 4 H), 6.85-6.81 (m, 2 H), 3.53-3.46 (m, 2 H), 3.15-3.07 (m, 2 H), 2.71-2.51 (m, 4 H).

The ^{13}C-NMR spectra of PDIEH and PDIIN derivatives provided detailed insights into their structural and electronic characteristics. For PDIEH (refer to Figure 2(c)), the aromatic carbons of the perylene diimide core displayed chemical shifts predominantly in the region of 123–138 ppm, characteristic of extended π-conjugation and electron-deficient aromatic systems. Notably, the signals observed around 163.8 ppm corresponded to carbonyl groups, confirming the presence of the imide functionality.

^{13}C NMR of PDIEH (100 MHz, $CDCl_3$) δ (ppm): 163.81, 134.52, 131.47, 129.43, 126.39, 123.40, 123.12, 44.50, 38.13, 30.93, 28.87, 24.24, 23.24, 14.26, 10.79.

^{13}C NMR of PDIIN (100 MHz, $CDCl_3$) δ (ppm): 163.53, 144.22, 141.69, 131.65, 127.75, 126.47, 124.96, 123.21, 122.87, 57.25, 31.73, 29.05.

Additionally, alkyl-chain substituents were identified through prominent signals at around 14-44 ppm, consistent with aliphatic carbons. In contrast, PDIIN showed aromatic carbon signals in a similar range (122-144 ppm), but with slight shifts due to phenyl substituents (refer to Figure 2(d)). The aromatic phenyl groups were distinctly resolved with signals observed at 122–135

ppm. In comparison, the imide carbonyl carbon was prominently observed at approximately 163.5 ppm, affirming the successful introduction of phenyl groups at the imide positions.

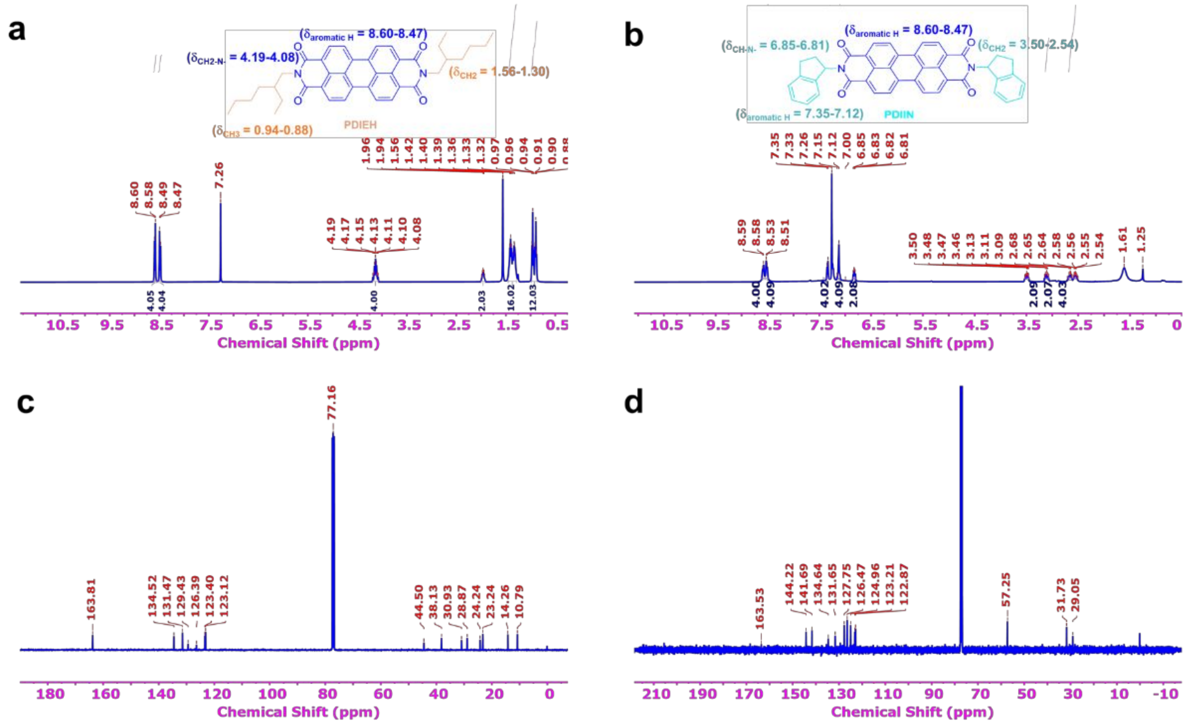

Figure S1 ^{1}H and ^{13}C NMR spectra of PDIEH (a) and (c) and PDIIN (b) and (d) recorded in $CDCl_3$. The ^{1}H NMR spectra confirm the presence of aromatic protons from the perylene core and aliphatic protons from side chains (PDIEH) or indane groups (PDIIN). The ^{13}C NMR spectra further validate the structural integrity of both compounds, showing characteristic peaks corresponding to aromatic and alkyl carbon environments. The chemical shifts are consistent with the expected molecular structures, confirming successful synthesis of the target non-fullerene acceptors.

The differences observed in chemical shifts between PDIEH and PDIIN underscore the structural modifications and their impact on electron distribution. This is crucial for understanding their influence on photovoltaic performance when blended with donor polymers.

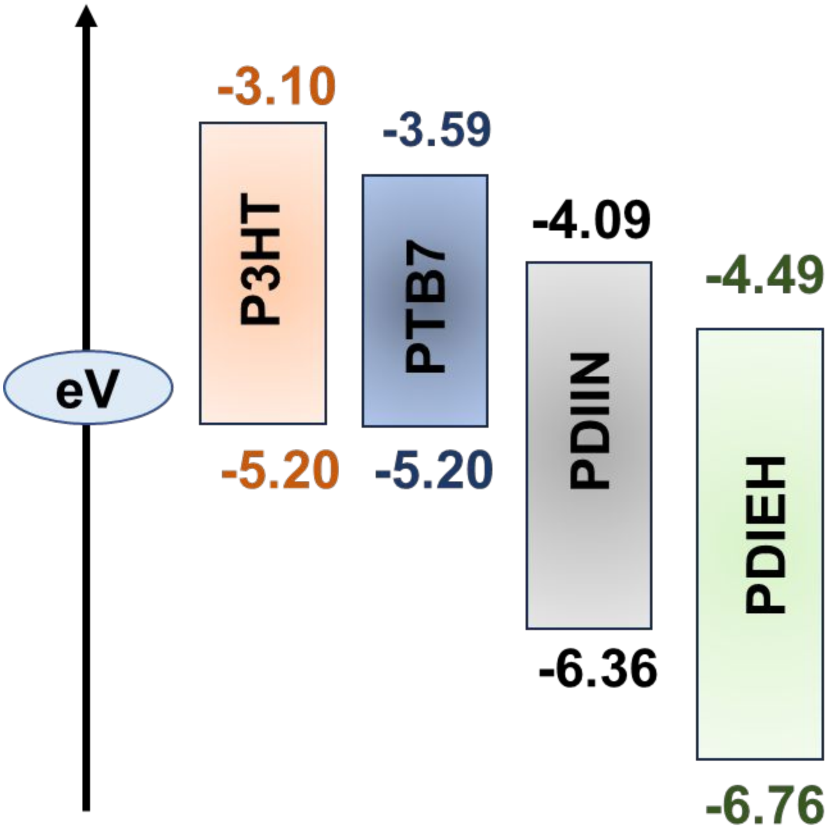

Figure S2. Energy level alignment of donor polymers (P3HT and PTB7) and non-fullerene acceptors (PDIIN and PDIEH), showing the HOMO and LUMO energy levels (in eV) derived from cyclic voltammetry and DFT calculations. The favorable LUMO offsets between the donors and acceptors facilitate efficient electron transfer. At the same time, the deeper LUMO level of PDIEH supports enhanced charge separation and reduced recombination in organic solar cells.

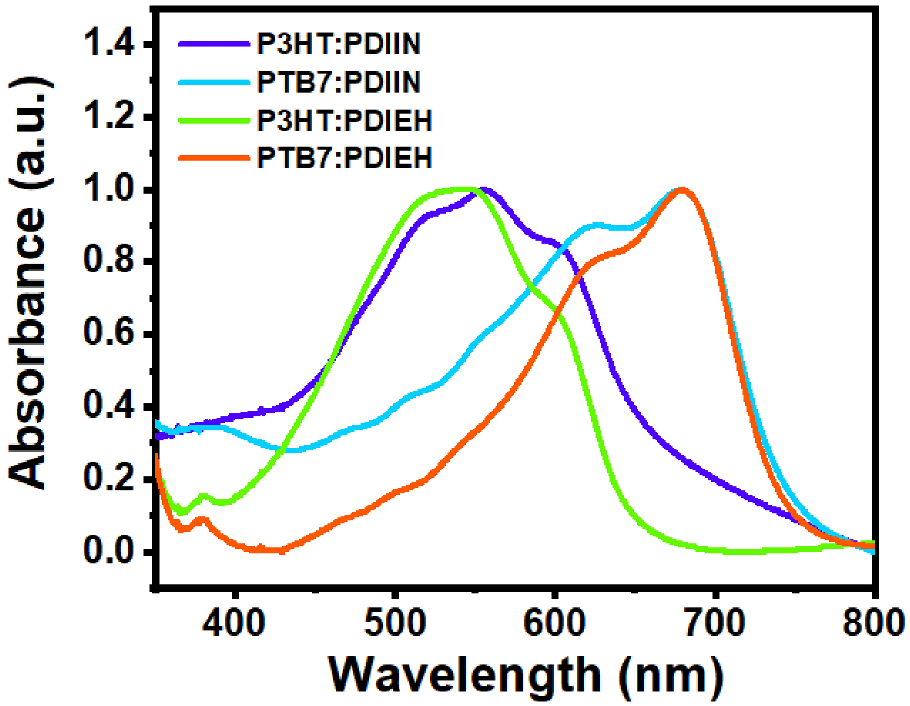

Figure S3: Steady-state absorption spectra of P3HT:PDIEH, P3HT:PDIIN, PTB7:PDIEH, and PTB7:PDIIN blends. UV-Vis spectra demonstrate redshift, indicating significant band alignment changes crucial for BHJ.

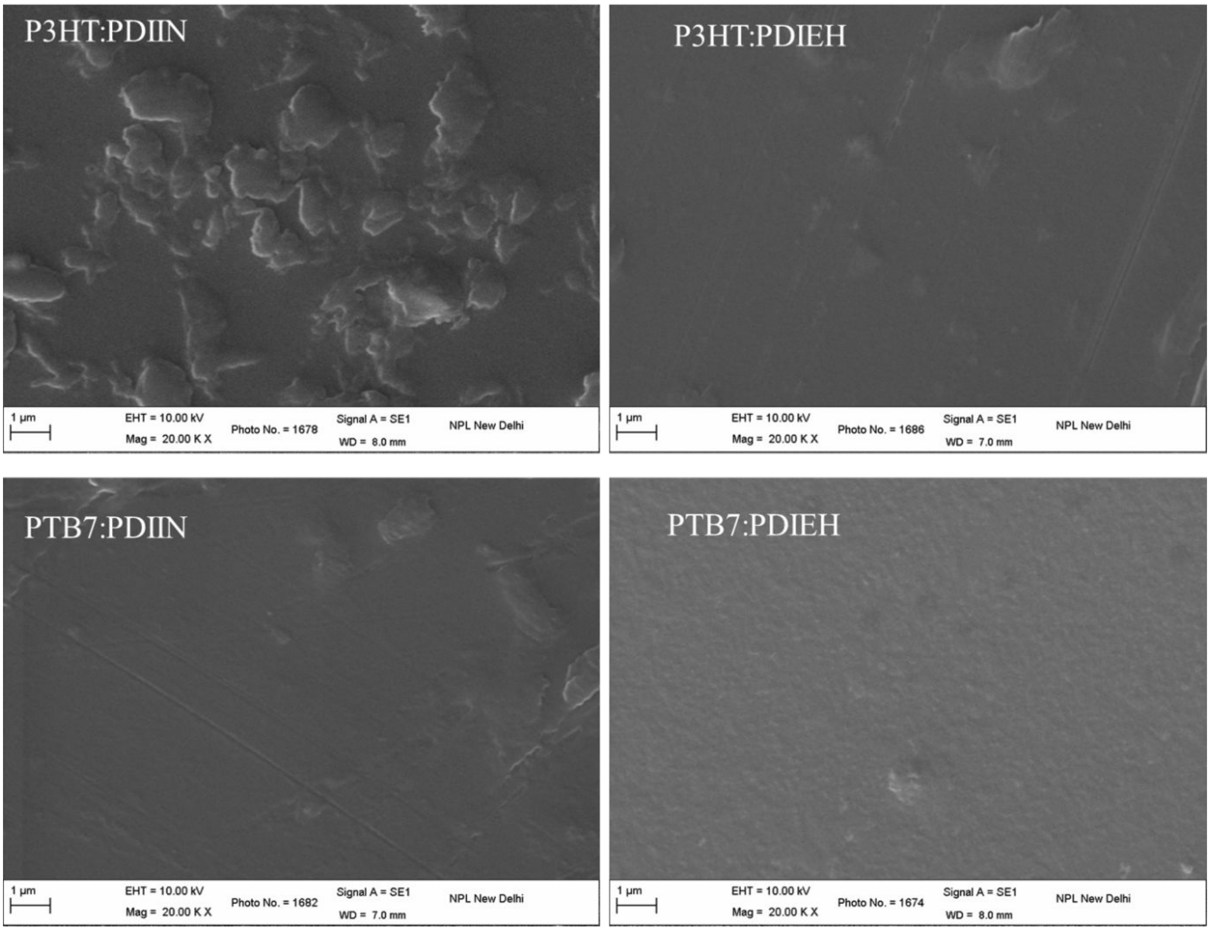

Figure S4: SEM micrographs of the four donor–acceptor blend films: P3HT:PDIIN, PTB7:PDIIN, P3HT:PDIEH, and PTB7:PDIEH. The images illustrate significant differences in film morphology driven by side-chain variation, where PDIEH blends exhibit smooth, uniform surfaces and PDIIN blends show pronounced roughness and domain features, indicating heterogeneous molecular packing.

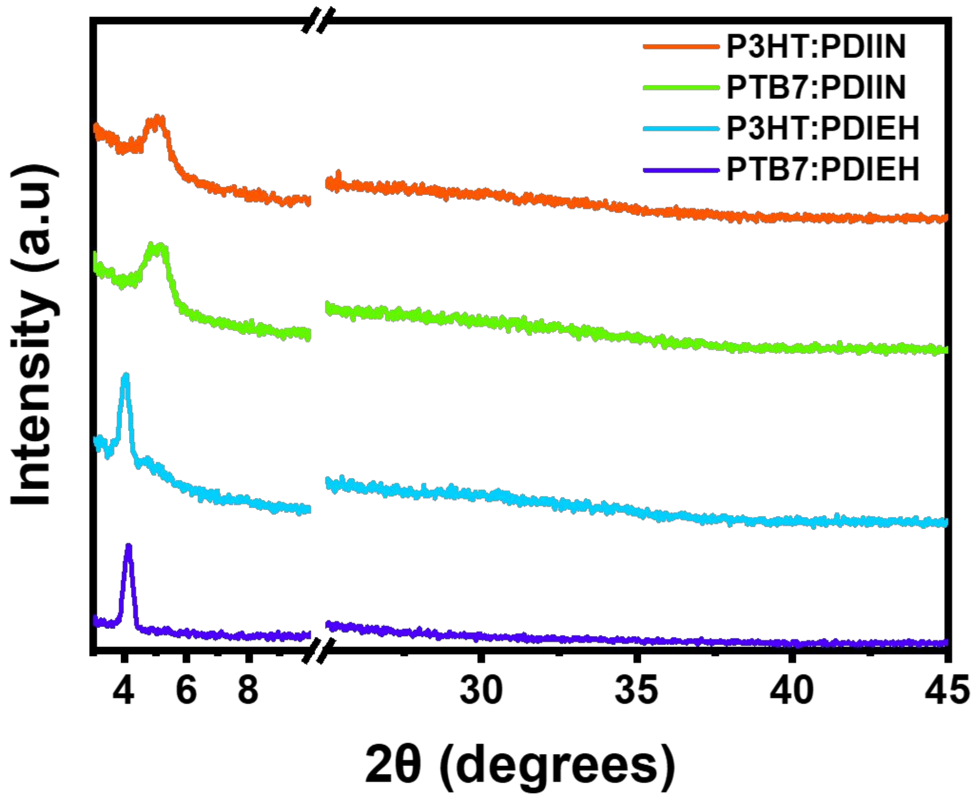

Figure S5: GIXRD patterns of P3HT:PDIIN, PTB7:PDIIN, P3HT:PDIEH, and PTB7:PDIEH blend films. The prominent peak at low 2θ2θ in PDIEH blends reflects a highly ordered arrangement of molecules, while the broader, less intense features in PDIIN blends indicate greater molecular disorder. These results confirm that side-chain engineering strongly affects blend crystallinity and molecular arrangement in solid-state films.

SAMPLE PREPARATION FOR UTAS

In preparing thin films for UTAS characterization, quartz substrates were initially subjected to a rigorous cleaning regimen. This involved sequential sonication in de-ionized water, acetone, and isopropanol, each for a duration of ten minutes, to ensure the removal of organic and inorganic contaminants.

The organic semiconductors PDIIN and PDIEH were separately solubilized in chlorobenzene to a 20 mg/mL concentration. These solutions were magnetically stirred at room temperature overnight to achieve homogeneity. Subsequently, the PDIIN and PDIEH solutions were deposited onto the pre-cleaned quartz substrates using a spin-coating technique at 1500 revolutions per minute (rpm) for 60 seconds.

Similarly, the semiconducting polymers P3HT and PTB7 were dissolved in chlorobenzene at an identical 20 mg/mL concentration and stirred overnight at room temperature. These solutions were then spin-coated onto the quartz substrates at a lower spinning rate of 1000 rpm for a duration of 60 seconds.

To fabricate blend films, mixtures of P3HT:PDIIN, P3HT:PDIEH, PTB7:PDIIN, and PTB7:PDIEH were prepared in chlorobenzene. Each mixture maintained a 1:1 weight ratio and a 20 mg/mL concentration. These blend solutions were then spin-coated onto the quartz substrates at 1000 rpm for 60 seconds.

Post-deposition, the films of PDIIN and PDIEH were subjected to an annealing process at 110°C for 10 minutes within a nitrogen-filled glove box to improve their morphological properties. Similarly, films composed of P3HT, PTB7, and the various blend compositions underwent an annealing process at 110°C for 15 minutes under a nitrogen atmosphere in a glove box.

UTAS CALCULATION

The Surface Xplorer software was utilized to calculate TAS time components. This process required fitting the decay kinetics for both the PIA and the GSB peak. Two fitting formulas were employed for this purpose. The first, a four-component fitting formula, is expressed as

$$\mathbf{y = y_0 + A_1exp(-x/\tau_1) + A_2exp(-x/\tau_2) + A_3exp(-x/\tau_3) + A_4exp(-x/\tau_4)} \quad \textbf{(1)}$$

The second, a three-component fitting formula, simplifies to

$$\mathbf{y = y_0 + A_1exp(-x/\tau_1) + A_2exp(-x/\tau_2) + A_3exp(-x/\tau_3)} \quad \textbf{(2)}$$

Carrier lifetime

For three-component fitting

$$\mathbf{\tau_{av} = (A_1\tau_2 + A_2\tau_2 + A_3\tau_2)/(A_1 + A_2 + A_3)} \quad \textbf{(3)}$$

For four-component fitting

$$\mathbf{\tau_{av} = (A_1\tau_2 + A_2\tau_2 + A_3\tau_2 +A_4\tau_4)/(A_1 + A_2 + A_3\ A_4)} \quad \textbf{(4)}$$

The parameters τ_1, τ_2, τ_3, and τ_4 represent the time taken by charge carriers to undergo relaxation processes, while the coefficients A_1, A_2, A_3, and A_4 represent the proportions of carriers undergoing relaxation via each process. The significance of these parameters varies depending on the material and peak position. In donor materials, a three-component fitting of decay kinetics is performed for GSB, where τ_1, τ_2, and τ_3 represent the short-lived species after excitation in which charge carriers exhibit a charge transfer state, carriers get trapped in trap states, and charge carriers undergo recombination, respectively. In acceptor materials, a three-component fitting of decay kinetics is performed for PIA, where τ_1, τ_2, and τ_3 represent the short-lived species after the PIA in which charge carriers show a charge transfer state or relax to a lower excited state, carriers get trapped in trap states, and charge carriers undergo recombination, respectively. In blends, a three-component fitting of decay kinetics is performed for PIA, where τ_1, τ_2, and τ_3 represent the relaxation of hot carriers, bounded polaron pair to separated polarons, and the recombination of charge carriers, respectively. In blends, a four-component fitting of decay kinetics is performed for GSB, where τ_1, τ_2, τ_3, and τ_4 represent the time constants related to charge transfer (CT), separated polarons (SP) (dissociated BPP), trapped BPP, and the recombination of charge carriers, respectively. Table S1 presents a comprehensive overview of the parameters, their respective significance, and material-specific characteristics crucial for the analysis of UTAS. The table encapsulates key details such as the time constants (τ_1, τ_2, τ_3, τ_4), their implications in various materials, and the corresponding peaks (GSB or PIA) for which the data is fitted.

Table S4: Summary of time constant parameters, significance, and terms used in UTAS Analysis.

Parameter/Term	Definition	Physical Interpretation
**Lifetime Constants**	τ_1, τ_2, τ_3, τ_4: Timescales determined from kinetic fitting	Charge transfer (τ_1), polaron separation (τ_2), trapping (τ_3), and recombination (τ_4), respectively.
**Kinetic Components**	A_1-A_4: Amplitude coefficients for each lifetime	Contribution of relaxation processes
**Ground-State Bleach**	GSB: Negative signal in TA spectrum	Depopulation of the ground state by photoexcitation
**Photoinduced Absorption**	PIA: Positive signal in TA spectrum	Formation/stabilization of polaron or exciton states

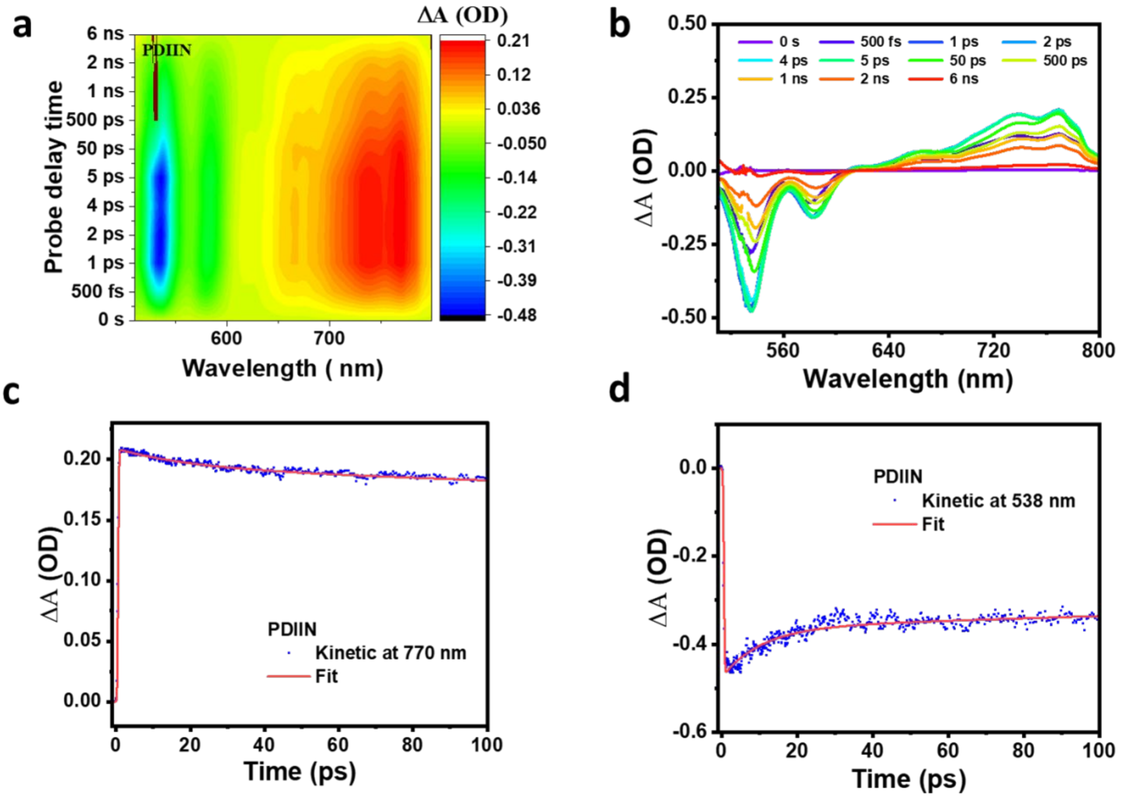

Figure S6: Characterization of Transient Dynamics in PDIIN. (a) 2D contour plot depicting transient absorption spectra, (b) Transient spectral profile ranging from 510 nm to 800 nm with pump excitation at 350 nm, (c) Transient kinetics decay of PDIIN at Photo Induced Absorption (770 nm), and (d) Transient kinetics decay of PDIIN at Ground State bleaching (538 nm). These subplots provide insights into the photoinduced processes and temporal evolution of NDIID dynamics.

Table S5: Kinetic fitting parameters for all blends at Ground State Bleaching (GSB), Photo Induced Absorption (PIA). τ_1, τ_2, and τ_3 represent the decay time constants associated with the charge transfer state or relaxation to a lower excited state, trapped BPP, and the recombination of charge carriers, respectively. The coefficients A_1, A_2, and A_3 represent the proportions of carriers undergoing relaxation via each process.

Material	Wavelength (nm)	A_1 (%)	τ_1 (ps)	A_2 (%)	τ_2 (ps)	A_3 (%)	τ_3 (ps)	τ_{avg} (ps)
**PDIIN (GSB)**	537	22.70	10.5	26.10	682	51.20	2320	1368.22
**PDIIN (PIA)**	769	7.39	20.5	15.30	563	77.30	3020	2422.11

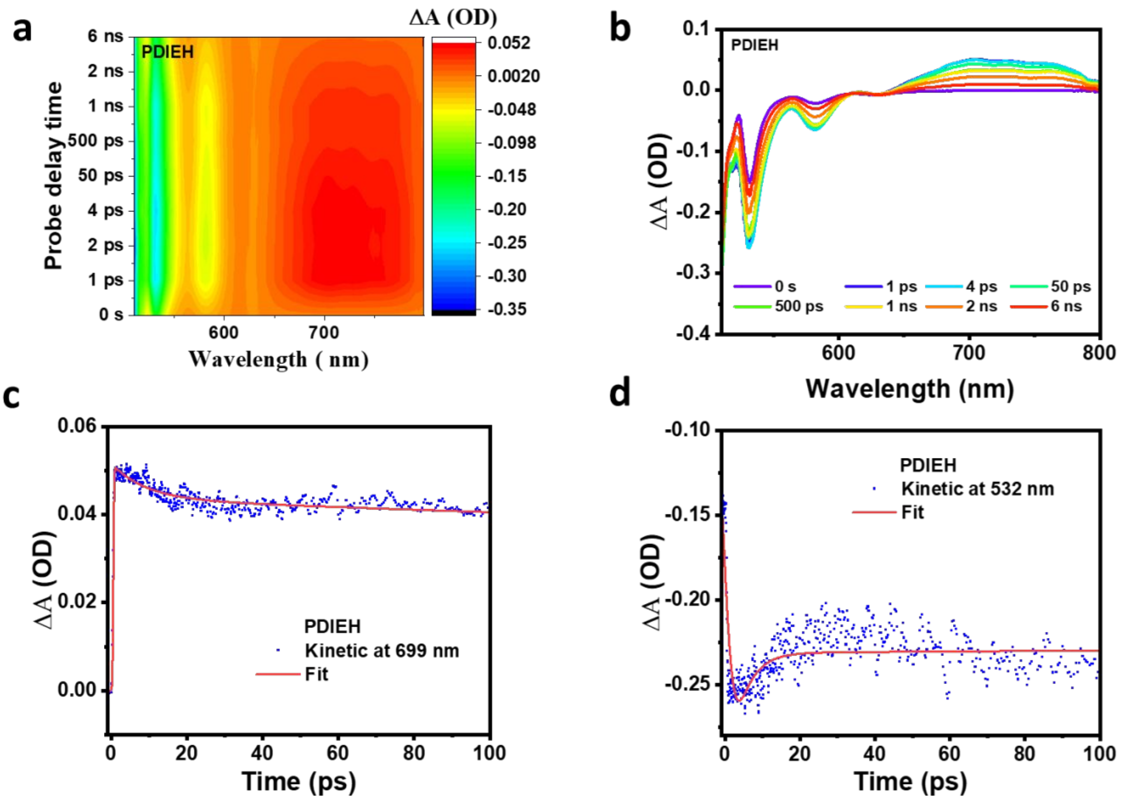

Figure S7*:* Characterization of Transient Dynamics in PDIEH. (a) 2D contour plot depicting transient absorption spectra, (b) Transient spectral profile ranging from 510 nm to 800 nm with pump excitation at 350 nm, (c) Transient kinetics decay of PDIEH at Photo Induced Absorption (699 nm), and (d) Transient kinetics decay of PDIEH at Ground State Bleaching (532 nm). These subplots provide insights into the photoinduced processes and temporal evolution of PCBM dynamics.

Table S6: Kinetic fitting parameters for all blends at Ground State Bleaching (GSB) Photo Induced Absorption (PIA). τ_1, τ_2, and τ_3 represent the decay time constants associated with the charge transfer state or relaxation to a lower excited state, trapped BPP, and the recombination of charge carriers, respectively. The coefficients A_1, A_2, and A_3 represent the proportions of carriers undergoing relaxation via each process.

**Material**	**Wavelength (nm)**	**A_1 (%)**	**τ_1 (ps)**	**A_2 (%)**	**τ_2 (ps)**	**A_3 (%)**	**τ_3 (ps)**	**τ_{avg} (ps)**
**PDIEH (GSB)**	532	34.40	4.21	65.60	> 6ns	-	-	> 6ns
**PDIEH (PIA)**	699	14.80	8.92	14.50	272	70.80	4350	3120.56

REFERENCE

S1 Gaussian 09, Revision C.01, M. J. Frisch, G. W. Trucks, H. B. Schlegel, G. E. Scuseria, M. A. Robb, J. R. Cheeseman, G. Scalmani, V. Barone, B. Mennucci, G. A. Petersson, H. Nakatsuji, M. Caricato, X. Li, H. P. Hratchian, A. F. Izmaylov, J. Bloino, G. Zheng, J. L. Sonnenberg, M. Hada, M. Ehara, K. Toyota, R. Fukuda, J. Hasegawa, M. Ishida, T. Nakajima, Y. Honda, O. Kitao, H. Nakai, T. Vreven, J. A. Montgomery, Jr., J. E. Peralta, F. Ogliaro, M. Bearpark, J. J. Heyd, E. Brothers, K. N. Kudin, V. N. Staroverov, T. Keith, R. Kobayashi, J.Normand, K. Raghavachari, A. Rendell, J. C. Burant, S. S. Iyengar, J. Tomasi, M. Cossi, N.Rega, J. M. Millam, M. Klene, J. E. Knox, J. B. Cross, V. Bakken, C. Adamo, J. Jaramillo, R. Gomperts, R. E. Stratmann, O. Yazyev, A. J. Austin, R. Cammi, C. Pomelli, J. W. Ochterski, R. L. Martin, K. Morokuma, V. G. Zakrzewski, G. A. Voth, P. Salvador, J. J. Dannenberg, S.Dapprich, A. D. Daniels, O. Farkas, J. B. Foresman, J. V. Ortiz, J. Cioslowski, and D. J. Fox, Gaussian, Inc., Wallingford CT, 2010.
